# Supplementary material for: Competition in Abies kawakamii forests at subtropical high mountain in Taiwan
Source: PLoS One. 2021 Jul 22;16(7):e0254791. doi: 10.1371/journal.pone.0254791 (PMC8297911; doi:10.1371/journal.pone.0254791)
Supplement: Attachment — Submitted filename: Response to Reviewers_20210523.docx [file pone.0254791.s002.docx]

**Reviewer #1**

**Q1. Authors used DBH and inter-tree distance to compute the index, disregarding other tree attributes, such as crown dimensions and tree heights.**

Reply to Q1. We add competitive index (CI) count by crown volume (CV) (Line 136-140, 160-163), but we suffer dilemma that we still use CI computed by DBH (Line 194-201).

**Q2. Authors used the fixed search radius approach for finding the competitor around the subject tree, which is less robust approach and it should have been used with some improvements, such as variable radius approach.**

Reply to Q2. Thanks to your comment, we revised our data and count CI by variable radius (D2), and we got better results in this article (Line154-159, 185-192, 205-212) we use CI-D2 to talk over the following chapters.

**Q3. Based on the methodological weakness of the study as main problem, and other problems, such as fewer sample plots, and poorly formulated texts including incomplete coverage of relevant literature studies and citations, lack of data summary statistics.**

Reply to Q3. We modified our description of the method & analysis (Line 130-180). We also add more relevant literature studies and citations (References 6, 19, 20, 27-30, 32-34, 38-41, 43, 45, 46). Moreover, we list more detailed information about data summary statistics (Table1-7).

**Reviewer #2**

**Q1. The Introduction is generally OK, but no strong explanation has been provided on why the study was conducted in the first place.**

Reply to Q1. Thank your comment, we revised the manuscript to add a description of this manuscript's importance (Line 74-79).

**Q2. What was the study period? Please specify, or otherwise delete. Add one sentence that will describe the number and the size of plots (in m2 or ha).**

Reply to Q2. We revised the manuscript to add the description of the study period and sample size in Line 116 & 123.

**Q3. In the section Results, whenever you describe any correlation, please add corresponding R2 values, not only descriptive text. You mention here that dead individuals exhibited a higher competitive index compared to the living individuals. But is this statistically significant? If so, provide computed p-value of the applied statistical test.**

Reply to Q3. Thank you for your comments. We revised all the tables which list *P* value & R^2^ (Table 4-7). Even though we compute competitive index with a variable radius (D2), dead individuals still have more stress than living individuals.
