# Supplementary material for: Competition in Abies kawakamii forests at subtropical high mountain in Taiwan
Source: PLoS One. 2021 Jul 22;16(7):e0254791. doi: 10.1371/journal.pone.0254791 (PMC8297911; doi:10.1371/journal.pone.0254791)
Supplement: Attachment — Submitted filename: Response to Reviewers_R2.docx [file pone.0254791.s003.docx]

The authors appreciate reviewers’ efforts and constructive comments. We have carefully considered all of the comments and thoroughly revised the manuscript. All revisions were highlighted in the revised manuscript, and a list of point-to-point responses was prepared in the following.

**Comment from reviewer 1:**

- **Comment 1.** *What’s stress resources, SSAs, woods, ethnic group?*

**Response:** Thanks for the reviewer's comment. Those mentioned errors, we seriously have inspected the article structure and modified the most unclear sentence and technical words. We also improve the consistency of technical words and linguistic revised in the manuscript. For example, competitive index was revised to competition index. Redundant phrases are already deleted and reduce the description. We marked the track changes with red font in the revised manuscript.

- **Comment 2.** *Please also state hypotheses/questions that you would evaluate or answer in your study.*

**Response:** Thanks for the reviewer’s comment. We state the five hypotheses about this study (Line 78-83) and the conclusion (Line 356-364) in the manuscript.

- **Comment 3.** *However, authors did not improve the writing style and wording, and due to this, I am not convinced that the manuscript of the current version is still not suitable for publication.*

**Response:** Thanks for the reviewer’s comment. We deeply and sincerely modified the writing style of the manuscript and improved our description of data analyses (Line 135-176). We also add more relevant literature studies and citations (References 42-43, 47-48) to prove us assume.

**Comment from reviewer 2:**

- **Comment 1.** *You have properly responded to all of my previous requests and concerns. The only thing that might be corrected before publication is the small part of the text that you have added in the process of revision. I have no further suggestions or requests.*

**Response:** Thanks for the reviewer’s affirmation.
